# Redox properties of extracellular polymeric substances (EPS) from electroactive bacteria

**DOI:** 10.1038/srep39098

**Published:** 2016-12-19

**Authors:** Shan-Wei Li, Guo-Ping Sheng, Yuan-Yuan Cheng, Han-Qing Yu

**Affiliations:** 1 CAS Key Laboratory of Urban Pollutant Conversion, Department of Chemistry, University of Science and Technology of China, Hefei, 230026, China

## Abstract

Although the capacity for electroactive bacteria to convert environmental metallic minerals and organic pollutants is well known, the role of the redox properties of microbial extracellular polymeric substances (EPS) in this process is poorly understood. In this work, the redox properties of EPS from two widely present electroactive bacterial strains (*Shewanella oneidensis* and *Pseudomonas putida*) were explored. Electrochemical analysis demonstrates that the EPS extracted from the two strains exhibited redox properties. Spectroelectrochemical and protein electrophoresis analyses indicate that the extracted EPS from *S. oneidensis* and *P. putida* contained heme-binding proteins, which were identified as the possible redox components in the EPS. The results of heme-mediated behavior of EPS may provide an insight into the important roles of EPS in electroactive bacteria to maximize their redox capability for biogeochemical cycling, environmental bioremediation and wastewater treatment.

Extracellular polymeric substances (EPS) are a complex high-molecular-weight polymer mixture composed mainly of proteins^1^, polysaccharides^2^, humic substances, etc., that are secreted by microorganisms^3^. The presence of EPS in pure and mixed cultures has been reported^4^,^5^. Because of their important roles in the biogeochemical cycling of pollutants (e.g., biosorption, biomineralization, and biocorrosion^6^), microbial EPS have attracted extensive attention. While few studies have been performed on the redox features of EPS because their complex and heterogeneous compositions and characteristics make this topic of research difficult to investigate. It has been suggested that the redox properties of EPS may arise from bacterial refractory polymers, such as proteins and, possibly, humic substances, which could serve as electron donors or acceptors in bacterial biofilms^7^.

Electroactive bacteria, which can transport electrons over biological membranes to or from their extracellular environment, have been successfully used to generate electricity from waste materials^8^,^9^. The redox properties of EPS in electroactive bacteria may be important to their ability to biochemically modify metals and organic pollutants. EPS extracted from a model electroactive bacterium, *Shewanella,* has been shown to be involved in U(VI) immobilization through sorption and reduction of U(VI)^4^,^10^. Additionally, EPS extracted from *Shewanella* may enhance the biotransformation of organic pollutants^11^,^12^,^13^. Recently, approximately 20 redox proteins in EPS from *Shewanella* sp. were identified^14^. Though there is limited research into the redox properties of *Pseudomonas putida* EPS, some studies have reported that *P. putida* can reduce arsenate^15^ and biotransform dibenzothiophene^16^. These results suggest that the redox properties of EPS play important roles in the migration and transformation of redox-sensitive contaminants by electroctive bacteria. It has also been reported that EPS extracted from *Escherichia coli* could reduce positively charged silver ions to silver nanoparticles^17^. However, the redox properties and the possible redox components of EPS from various electroactive bacteria are not well documented.

In this study, the redox properties of EPS from two widely present electroactive bacterial strains (*S. oneidensis*^18^ and *P. putid*a^19^), and the relationships between the compositions of these EPS and their redox properties were investigated. The results of this work provide an insight into the relationship between microbial extracellular electron transfer and the redox properties of EPS from electroactive bacteria.

## Results and Discussion

### EPS components and structure analysis

**Figure 1:**
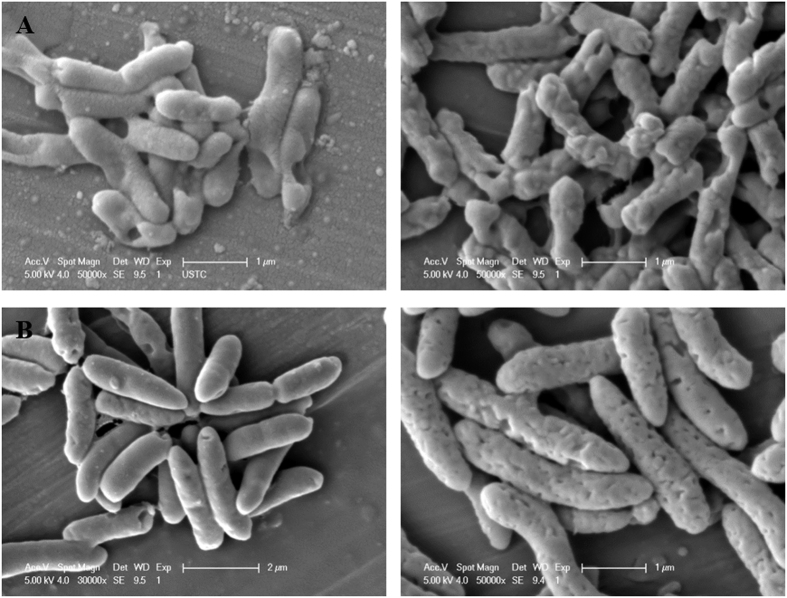
SEM images of cells of two electroactive bacteria before (left) and after (right) EPS extraction: (**A**) *S. oneidensis* and (**B**) *P. putida*.

EPS of two electroactive bacteria were extracted by EDTA method and the feasibility of the extraction method was confirmed by SEM photographing before and after EPS extraction (Fig. 1). Proteins and polysaccharides were found to be the main components of the two electroactive bacterial EPS (Table 1). The EPS of *S. oneidensis* and *P. putida* contained more proteins, including heme-binding proteins. Uronic acids was the main component in the polysaccharides of the two EPS samples, and the average content of uronic acids in polysaccharides of each EPS was about 42%.

The structure and functional groups of EPS were analyzed using FTIR spectroscopy (Figure S1). Table S1 listed the detailed peak assignments of FTIR spectra of EPS, which were referred to the previous reports^20^,^21^. The bands of amide I (1700–1600 cm^−1^), amide II (1600–1500 cm^−1^) and amide III (1350–1250 cm^−1^) were attributed to proteins, while C-O (1200–1000 cm^−1^) was associated with polysaccharides. The amide I band at 1636 cm^−1^ was generally ascribed to C=O stretching vibrations of β–sheet of proteins. The band at 1259 cm^−1^ was the representative amide III band, which is attributed to N-H bending and C-N stretching in proteins. The broad peak at 3427 cm^−1^ was attributed to O-H stretching. The bands at 2925 cm^−1^ and 2853 cm^−1^ were assigned to C-H stretching of methylene group in fatty acids. The band at 1408 cm^−1^ was ascribed to symmetric deformation of CH_3_ and CH_2_ of proteins and symmetric C-O stretching of carboxylic groups. The bands at 1086 cm^−1^ and 1043 cm^−1^ was attributed to the C-O bond, the C-O-C bond and the C-H bond from polysaccharides. The band at 1086 cm^−1^ was also assigned to the asymmetric and symmetric P=O stretching of the phosphodiester backbone of nucleic acids.

### Redox Properties of EPS

**Figure 2:**
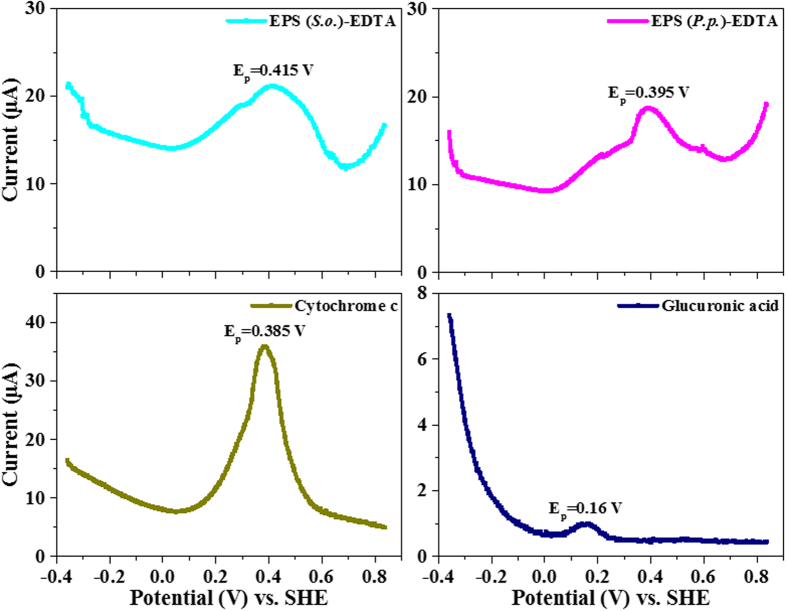
Differential pulse voltammetry of EPS extracted from *S. oneidensis* and *P. putida* by using EDTA method, bovine heart cytochrome c, and glucuronic acid.

Differential pulse voltammetry (DPV) was applied to identify the redox properties of the two electroactive bacterial EPS. Simultaneously, the electrochemical activities of bovine heart cytochrome c and glucuronic acid were measured as references. Figure 2 shows that the voltammograms of two EPS solution all exist an obvious oxidation peak. The DPV peak potentials of bovine heart cytochrome c, *S. oneidensis* EPS and *P. putida* EPS are 0.385 V, 0.415 V and 0.395 V (vs. standard hydrogen electrode, SHE), respectively. It shows that the DPV peak potential of bovine heart cytochrome c was similar to those measured in EPS samples. However, glucuronic acid only exhibited a weak redox peak. These results imply that two electroactive bacterial EPS all existed redox matters, and cytochrome c might be the main redox matter in EPS.

In order to investigate the effects of various EPS extraction methods on the redox properties of electroactive bacterial EPS, three methods (i.e., heating, cation exchange resin and EDTA methods) were used, and the redox properties of the extracted EPS were compared (Figs 2 and S2). Results show that two electroactive bacterial EPS extracted by the heating and EDTA methods had similar DPV peak potentials with high peak currents, while the cation exchange resin extraction method had a great change on the DPV peak potentials of two electroactive bacterial EPS with small peak currents. This implied that the EPS extracted from the three methods were all electrochemical-activated, and extraction methods influenced the redox properties of the bacterial EPS.

### Redox Components in EPS

**Figure 3:**
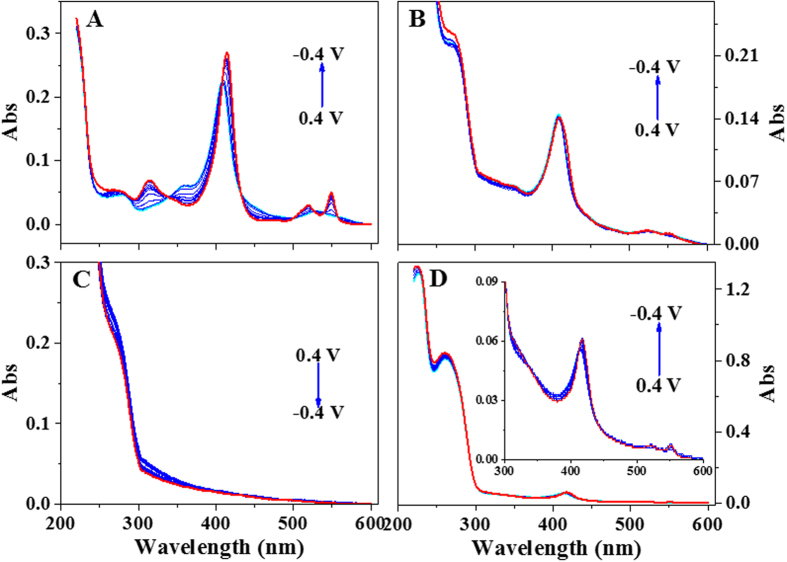
UV-Vis spectroelectrochemical analysis of EPS of the three bacterial strains and bovine heart cytochrome c. (**A**) bovine heart cytochrome c, (**B**) EPS of *S. oneidensis*, (**C**) EPS of *E. coli*, and (**D**) EPS of *P. putida*. The cyan curve and the red curve at each figure represented the origin sample and the reduced sample at reduction voltage of −0.4 V.

EPS were reduced at an applied negative potential, and the spectra changes of the EPS after reduction could be monitored using a spectroelectrochemical method. The UV-Vis spectra of the EPS solutions after electrochemical reduction at various negative potentials are shown in Fig. 3, the EPS of *E. coli* was used as a negative control with 10 g/L. Figure 3A shows that bovine heart cytochrome c at its native oxidation state showed an oxidation peak at 529 nm, and after negative potentials were applied, two reduction peaks appeared at 519 nm and 549 nm. The absorbance ratio at the two peaks (A_549_/A_519_) was related to the purity ratio of the reduced cytochrome c^22^, which was 1.7 at an applied potential of −0.4 V (vs. SHE). The spectra of EPS from *S. oneidensis* and *P. putida* (Fig. 3B and D) were similar to the spectrum of cytochrome c, as the same reduction peaks could be observed. The A_547_/A_524_ cytochrome purity ratio for the reduced *S. oneidensis* EPS was 0.81, and the A_551_/A_519_ ratio for the reduced *P. putida* EPS was 1. However, there were no absorbance peaks could be observed in the spectra of the EPS from a non-electroactive bacterium, *E. coli* (Fig. 3C).

**Figure 4:**
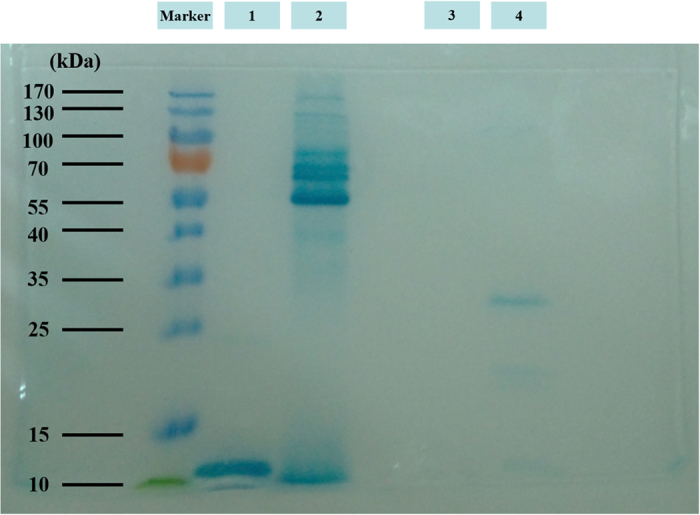
LDS-PAGE of EPS of the three bacterial strains and bovine heart cytochrome c with heme staining. 1: bovine heart cytochrome c, 2: EPS of *S. oneidensis*, 3: EPS of *E. coli*, and 4: EPS of *P. putida*.

Cytochrome c may exist in the EPS from electroactive bacteria and contribute to its redox properties. To validate this hypothesis, a heme-binding-protein LDS-PAGE electrophoresis was conducted to determine if heme-binding proteins were present in the EPS of two electroactive bacteria, the EPS of *E. coli* was used as a negative control with 6 g/L. As shown in Fig. 4, the LDS-PAGE of the three EPS with heme staining confirms that the EPS from *S. oneidensis* and *P. putida* contained heme-binding proteins. Furthermore, the EPS of *S. oneidensis* contained more types of heme-binding proteins than *P. putida*. The molecular weights of most of the electrophoretic bands of the *S. oneidensis* EPS ranged from 55 to 100 kDa, and those of *P. putida* EPS were mainly <35 kDa. However, there were no detected heme-binding proteins in the non-electroactive bacterium *E. coli* EPS. The cytochrome c species MtrC and OmcA in *S. oneidensis* outer membrane mediate extracellular redox reactions, and their molecular weights are 71.3 kDa and 78.7 kDa, respectively^14^. These molecular weights are in the range of 55 to 100 kDa shown in the electrophoresis image of *S. oneidensis* EPS (Fig. 4), suggesting that these two types of cytochrome c may exist in *S. oneidensis* EPS. This finding is in agreement with previous research, in which the two c-type cytochromes were also identified in EPS extracted from *Shewanella* sp. HRCR-1 by proteomics analysis^14^. The molecular weights of the electrophoretic bands of *P. putida* EPS (<35 kDa) were similar to the bands of membrane fractions of spheroplasts from *Pseudomonas stutzeri*^23^. This band interval of 25.6–19.7 kDa may be related to the di-heme protein cytochrome c_4_^22^, a membrane-bound bacterial electron transfer cytochrome found in *Pseudomonas aeruginosa*^24^, *P. stutzeri*^25^ and *Shewanella baltica*^26^ that is involved in the electron-transport systems associated with both aerobic and anaerobic respiration. It has also been reported that the A_551_/A_522_ ratio of cytochromes c_4_ from *P. aeruginosa* and *P. stutzeri* were 1.36 and 1.21, respectively^27^, which were similar to the ratio of 1 found in *P. putida* EPS. Heme-binding-protein electrophoresis illustrates that heme-binding proteins of various molecular weights and species are present in the EPS from *S. oneidensis* and *P. putida*.

The spectroelectrochemical results and heme-binding-protein electrophoresis images suggest that membrane-bound heme-binding proteins may exist in the EPS isolated from *S. oneidensis* and *P. putida* and are likely the redox-active components accepting electrons through electrochemical reduction. These data suggest that heme-binding proteins are important redox components mediating electron transfer in electroactive bacteria and other environmental microorganisms, as cytochrome c that contain heme is an important class of enzymes that participate in the respiratory chain. In natural environments, electroactive bacteria can use iron or manganese oxides and hydroxides as terminal electron acceptors to generate energy for biosynthesis and cell maintenance. EPS can facilitate the attachment of microorganisms to the mineral surface, and the EPS layer comprises several redox species to enhance attacking the mineral surface through an electrochemical dissolution^6^. Thus, electroactive bacterial EPS mediate their direct contact with electron acceptors to accelerate extracellular electron transfer for mineral biotransformation^28^.

## Conclusions

In summary, heme-binding proteins were identified as the key redox species in the EPS of *S. oneidensis* and *P. putida* for electroactive bacterial extracellular electron transfer. Heme-binding proteins in EPS of electroactive bacteria are usually able to transfer electrons between membrane-bound reduction systems and terminal oxidases. Thus, exploring the redox properties of electroactive bacterial EPS could provide in-depth understanding of the interactions between electroactive bacteria and external electron acceptors, such as metals, organic contaminants, and insoluble minerals, which is important for understanding microbial extracellular electron transfer. So, establishing the relationships between electroactive bacterial EPS and the process of extracellular electron transfer and developing the methods to discover other potential redox species in electroactive bacterial EPS continue to pose challenges for the field.

## Methods

### Bacterial Strains

Wild-type *S. oneidensis* MR-1 (ATCC^®^ number 700550^TM^) was kindly provided by Prof. K. H. Nealson from the University of Southern California^29^. *P. putid*a (CCTCC^®^ number AB2015303^TM^) was isolated through a photometric high-throughput method^30^ for electroactive bacteria screening from the lab-scale sequencing batch reactor that treated municipal wastewater.

### EPS Extraction

*S. oneidensis* and *P. putid*a were cultivated at 30 °C for 22 h in Luria-Bertani medium. The dispersed biomass was harvested by centrifugation at 5000 *g* for 5 min. EPS were isolated from these bacterial cells by an EDTA method, as previously described^14^. The dispersed cells were resuspended in 0.9% NaCl. An equal volume of 2% Na_2_-EDTA in 0.9% NaCl (pH 7.0) was mixed with the cell suspension and the mixture was incubated at 4 °C for 3 h. The cells were pelleted by centrifugation with 5000 *g* at 4 °C for 20 min, then the supernatant was filtered through 0.22 μm membrane filter. EPS were purified by ultrafiltration using a cellulose membrane (Millipore Inc., USA) with a molecular weight cut-off of 1 kDa. The EPS solution was then lyophilized to obtain pristine EPS and stored at −20 °C before use. The morphology of cell surfaces was imaged using a scanning electronic microscope (SEM, SIRION200, FEI Co., Netherlands) to investigate the microbial cell integrity before and after EPS isolation.

In order to compare the redox property of EPS extrated from different methods, heating and cation exchange resin (CER) methods were also used. EPS extraction by heating referred a previous report^31^. The dispersed biomass was harvested by centrifugation at 5000 *g* for 5 min. The bacterial pellet was re-suspended into 0.9% NaCl solution, and then was heated to 60 °C in a water bath for 30 min. After that, the solution was centrifuged at 5000 *g* for 15 min and then was filtrated. EPS extraction by CER was conducted according to a reported method^32^. The bacterial suspension was transferred to an extraction beaker and the CER (Dowex, 20–50 mesh in the sodium form, Sigma, USA) was added. The suspension was stirred at 300 rpm for 3 h at 4 °C. After that, the CER was removed and the suspension was centrifuged for 15 min at 5000 *g* and 4 °C in order to remove remaining bacterial cells. The supernatant was filtrated through 0.22 μm membrane and used as EPS solution.

### Chemical Analysis

The protein contents of the EPS were measured using a bicinchoninic acid (BCA) procedure^33^ with bovine serum albumin as the standard. Polysaccharides were determined using the anthrone method^33^ with glucose as the standard. Uronic acids were measured using the *m*-hydroxydiphenyl sulfuric acid method^34^ with glucuronic acid as the standard. Heme was quantified in a fluorescence assay^35^ with bovine heart cytochrome c (95%, Aladdin Co., USA) as the standard. DNA was detected by the diphenylamine colorimetric method^32^ with calf thymus DNA as the standard. The infrared spectra (IR) of EPS samples were obtained with a VERTEX 70 FTIR spectrometer (Bruker Co., Germany) to determine the functional groups in the EPS.

The electrochemical properties of the EPS were analyzed by DPV using an electrochemical workstation (660 C, Chenhua Instruments, Inc., China). The DPV measurements were performed with a 10 mL three-electrode cell with an Ag/AgCl reference electrode, a Pt counter electrode and an indium tin oxide working electrode. The parameters for DPV were: pulse increments 5 mV, pulse amplitude 50 mV, pulse width 300 ms, pulse period 5 s. The scanning range was from −0.4 V to 0.8 V vs. SHE and the scanning rate was 1 mV/s. Before measurements were taken, 10 mL blank solution (0.9% NaCl) were added to the three-electrode cell, and then the bovine heart cytochrome c (0.2 g/L), glucuronic acid (2 g/L) or the EPS solution was added to obtain a final concentration of 800 mg/L. The cell was purged with O_2_-free N_2_ for 15 min before electrochemical measurements. The curves of samples were deducted by the blank solution as background.

The UV-Vis spectra of the EPS at various potentials were measured using spectroelectrochemical analysis, which was performed using a thin-layer spectroelectrochemical cell with a Pt gauze working electrode, an Ag/AgCl reference electrode, and a Pt wire counter electrode^36^. The optical length of the spectroelectrochemical cell was 1 mm. To obtain a high resolution, a high concentration of EPS (10 g/L) was used for the spectroelectrochemical measurement. Bovine heart cytochrome c at 0.2 g/L was used as a reference. The sample volume in the spectroelectrochemical cell was 0.8 mL. The potential ranged from 0.4 V to −0.4 V vs. SHE, and the equilibration time at each potential was set to 15 min. The UV-Vis spectra of the EPS samples at each potential were then measured using a spectrophotometer (UV-2450, Shimadzu, Japan). The cell was purged with O_2_-free N_2_ for 15 min before electrochemical measurements and continued through the entire experiment to avoid the interference of oxygen.

### Electrophoresis of EPS Heme-Binding Proteins

Heme-binding protein electrophoresis was conducted to identify the presence of this redox component in the EPS, which was confirmed by lithium dodecyl sulfate (LDS)-PAGE and staining with tetramethylbenzidine (TMBZ, a chromogen that specifically binds to proteins with heme-associated peroxidase activity)^37^,^38^. Before analysis, the EPS samples were dissolved in deionized water, and the final concentration was adjusted to 6 g/L. Bovine heart cytochrome c (0.04 g/L) was used as control.

## Additional Information

**How to cite this article**: Li, S.-W. *et al*. Redox properties of extracellular polymeric substances (EPS) from electroactive bacteria. *Sci. Rep.* **6**, 39098; doi: 10.1038/srep39098 (2016).

**Publisher's note:** Springer Nature remains neutral with regard to jurisdictional claims in published maps and institutional affiliations.

## Supplementary Material

Supporting Information

## Figures and Tables

**Table 1:** The main compositions of three bacterial EPS.

Bacterium	Proteins (mg/L)	Polysaccharides (mg/L)	Uronic acids (mg/L)	Heme-binding proteins (mg/L)	DNA (mg/L)
*S. oneidensis*	9.14	4.52	2.45	1.84	0.40
*P. putida*	36.66	4.69	2.40	1.38	0.38
*E. coli*	7.25	4.34	2.35	0.45	0.29
