# Supplementary material for: Redox properties of extracellular polymeric substances (EPS) from electroactive bacteria
Source: Sci Rep. 2016 Dec 19;6:39098. doi: 10.1038/srep39098 (PMC5171820; doi:10.1038/srep39098)
Supplement: Supporting Information [file srep39098-s1.doc]

**Supporting Information**

**Redox properties of** **extracellular polymeric substances (EPS) from electroactive bacteria**

Shan-Wei Li, Guo-Ping Sheng^*^, Yuan-Yuan Cheng, Han-Qing Yu

CAS Key Laboratory of Urban Pollutant Conversion, Department of Chemistry, University of Science and Technology of China, Hefei, 230026, China

***Corresponding author:**

Prof. Guo-Ping Sheng

Fax: +86-551-63601592

E-mail: gpsheng@ustc.edu.cn


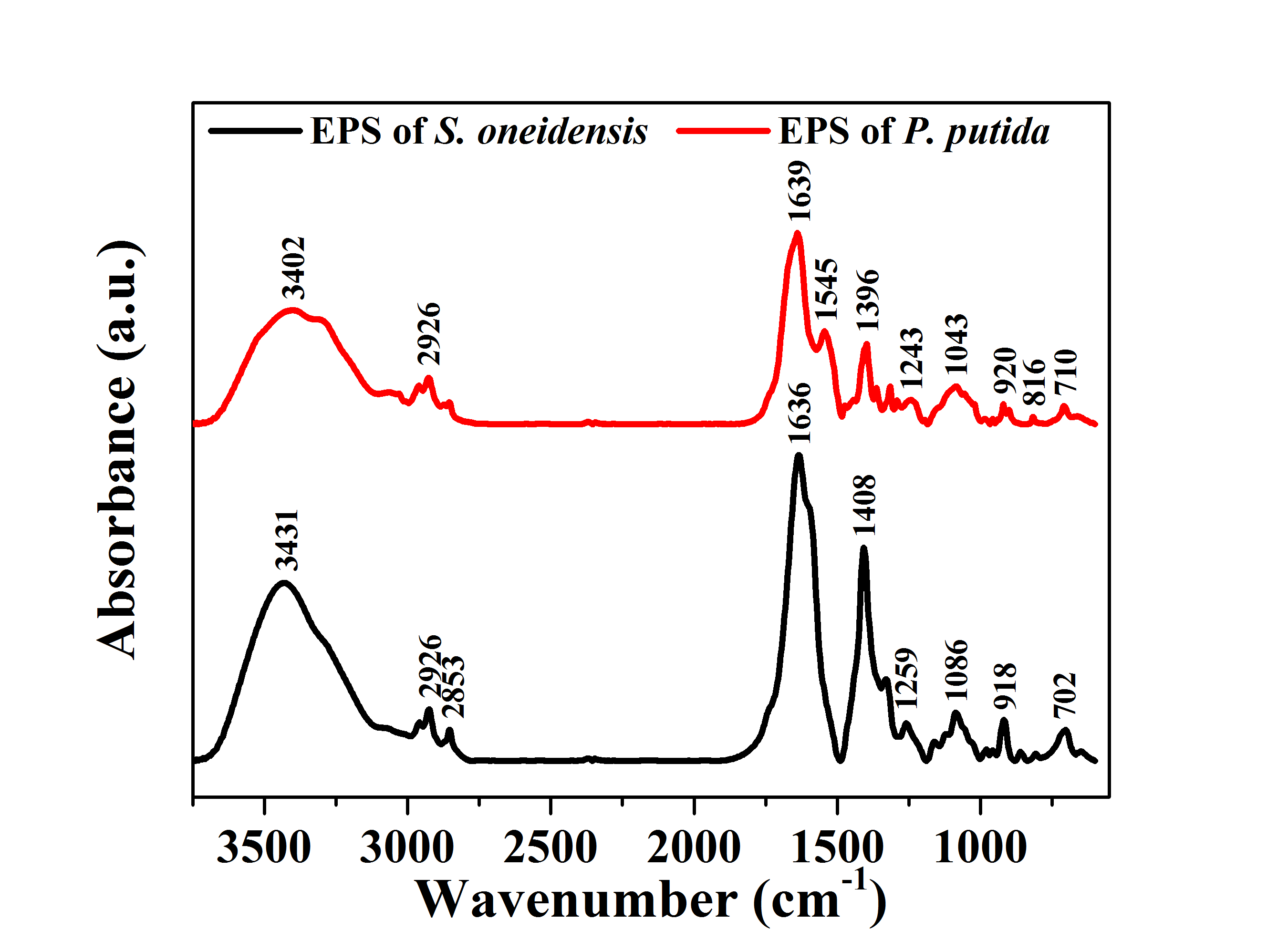


**Figure S1.** FTIR spectra of EPS isolated from two electroactive bacteria.

**Figure S2.** Voltammograms of EPS from two electroactive bacteria extracted by various extraction methods.

**Table S1. Peak assignment in the FTIR spectra of EPS.**

| **Wavenumber (cm^-1^)** | **Assignment** | **Functional groups** | **Note** |
| --- | --- | --- | --- |
| 3427 | O-H |  | Associate hydrogen bond |
| 2925/2853 | C-H | Lipids | Methylene group in fatty acids |
| 1636 | C=O | Proteins | Amide Ⅰ (β-sheet) |
| 1408 | CH_3_, CH_2_  C-O | Protein/Lipids  Carboxylic acids |  |
| 1259 | N-H, C-N | Proteins | Amide III |
| 1086, 1043, 1086 | C-O, C-O-C, C-H  P=O | Polysaccharides  Nucleic acids | Phosphodiester |
